# Ferritin Nanocages for Protein Delivery to Tumor Cells

**DOI:** 10.3390/molecules25040825

**Published:** 2020-02-13

**Authors:** Federica Palombarini, Elisa Di Fabio, Alberto Boffi, Alberto Macone, Alessandra Bonamore

**Affiliations:** Department of Biochemical Sciences “Alessandro Rossi Fanelli”, Sapienza University of Rome, Piazzale Aldo Moro 5, 00185 Rome, Italy; federica.palombarini@uniroma1.it (F.P.); elisa.difabio@uniroma1.it (E.D.F.); alberto.boffi@uniroma1.it (A.B.)

**Keywords:** ferritin, drug delivery, therapeutic proteins, nanocarrier, *Archaeoglobus fulgidus*, TfR1 receptor, tumor cells

## Abstract

The delivery of therapeutic proteins is one of the greatest challenges in the treatment of human diseases. In this frame, ferritins occupy a very special place. Thanks to their hollow spherical structure, they are used as modular nanocages for the delivery of anticancer drugs. More recently, the possibility of encapsulating even small proteins with enzymatic or cytotoxic activity is emerging. Among all ferritins, particular interest is paid to the *Archaeoglobus fulgidus* one, due to its peculiar ability to associate/dissociate in physiological conditions. This protein has also been engineered to allow recognition of human receptors and used in vitro for the delivery of cytotoxic proteins with extremely promising results.

## 1. Introduction

One of the major challenges in treating a range of human diseases is the use of therapeutic proteins and peptides. In fact, proteins can be used in enzyme replacement therapies or can exert cytotoxic activity towards mammalian cells. Venoms, plant toxins, microbial and animal toxins, antimicrobial peptides, proapoptotic proteins, and monoclonal antibodies (mAb) are the main sources of these kind of proteins. [1,2]. Venoms, for example, are able to induce cell-cycle alterations, apoptosis, necrosis, cellular membrane disruption, and cell membrane depolarization [3]. Many plant toxins, including saporin and ricin, are ribosome-inactivating proteins [4]. Animal toxins display an array of cytotoxic activity, ranging from membranolysis (melittin from *Apis mellifera*) [5] to chloride channels blocking (chlorotoxin from *Leiurus quinquestriatus*) [6]. Antimicrobial peptides, some of them known as anticancer peptides, promote cell lysis through pore formations [7]. Proapoptotic proteins, being of human origin, display a high clinical value in oncology, because they do not induce immunogenic responses [8]. mAb are the largest group of therapeutic proteins being able to inhibit specific receptors involved in cancer development [9].

Most protein drugs require efficient intracellular delivery to exert their therapeutic effects. The main drawback to their use is indeed the difficulty of delivering them inside the target cells due to their physicochemical properties, such as three-dimensional structure, hydrophilic/hydrophobic nature, and sensitivity to chemical modifications. For this reason, there is a growing interest in developing efficient and selective carriers or delivery systems [10,11,12,13,14,15].

From a very general point of view, drug delivery systems (DDS) are nanocarriers composed of lipids and/or polymers and their associated therapeutics. Over the years, several nanocarriers have been developed, including liposomes [16,17]; solid lipid nanoparticles (Compritol^®^ 888 ATO, Precirol^®^ ATO 5, Imwitor^®^ 900, etc.) [18,19]; polymeric nanoparticles (polylactide, chitosan, albumin, etc.) [20,21]; protein conjugates (antibody-drug conjugates and cell-penetrating peptide-drug conjugates) [22,23,24]; and viral capsids [25]. Many of these nanocarriers have been used, even successfully, to carry both traditional drugs and therapeutic proteins. In recent years, protein cages (hollow protein nanoparticles) have been taking center stage as DDS [26,27,28,29,30,31]. By “protein cage”, we mean a structure of protein nature consisting of subunits that self-assemble to form a highly symmetric hollow nanosphere large enough to enclose therapeutics such as conventional drugs, nucleic acids, and other proteins. In addition to viral capsids, excellent examples of protein cages are thermosomes [32], small heat-shock proteins [33], pyruvate dehydrogenase multienzyme complexes [34], and ferritins [35,36,37,38,39,40]. Although all these proteins have been extensively studied for the delivery of conventional drugs and/or contrast agents, very little has been done regarding their use in the delivery of therapeutic proteins. Most of the scientific literature in the field of protein drug encapsulation and delivery concern viral nanoparticles (VNPs), which, due to their highly symmetrical structures, can be considered one of the most versatile natural nanomaterials, although potentially infectious and hazardous in humans and other mammals. In this frame, a safer profile is shown by ferritins that, very recently, have been also tested for the delivery of therapeutic proteins [41,42]. Furthermore, ferritins have many other advantages as DDS: they can be produced at high yields in the form of recombinant proteins and are easily purified [43], they are biodegradable, and, due to their size, they can accumulate in highly vascularized tumor tissues (Enhanced Permeability and Retention (EPR) effect) [44].

Thus, this review will focus on the on-going research on ferritin-mediated protein drug delivery.

## 2. Ferritins as Modular Nanoplatforms for Drug Delivery

Ferritins are ubiquitous proteins found in all living organisms with the notable exception of yeast [45,46,47]. Their physiological function is linked to nonheme iron metabolism, being able to store up to 4500 iron ions per molecule. In fact, the name ferritin derives from the Latin “ferratus”, which means “covered with iron”. From a physiological point of view, it is important to keep Fe(III) protected within a protein shell to avoid cytoplasm iron overload with consequences in the normal biochemical processes related to this element. Indeed, high iron may cause increased production of reactive oxygen species (ROS) that can activate growth factor signaling pathways [48], as well as DNA mutations that eventually lead to tumor development.

**Figure 1:**
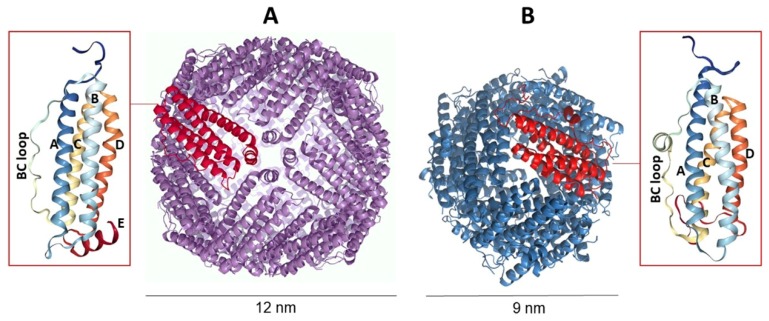
Comparison between canonical ferritin (**A**) and DNA-binding protein from starved cells (Dps) (**B**). While canonical ferritins form a cage of 24 subunits arranged in octahedral 4-3-2 symmetry, Dps proteins are dodecamers displaying 2- and 3-symmetry axes. The insets show the monomer structure consisting of a bundle of α-helices marked with capital letters.

Despite the very low sequence homology (in some cases, less than 20%), all ferritins share a common overall three-dimensional structure [49]. They can be grouped in three subfamilies, namely canonical ferritins (Ft), bacterioferritins (Bfr), and DNA-binding proteins from starved cells (Dps). Ft and Bfr proteins are classified as maxi-ferritins. They are composed of 24 subunits that assemble to form an octahedral hollow and spherical nanocage, with internal and external diameters of about 8 and 12 nm, respectively. Canonical Ft proteins are ubiquitous and share the same quaternary structure with Bfrs, which are typically found only in bacteria and archaea. The most significant difference between the Fts and Bfrs is the presence of twelve heme groups located at the subunit interfaces. Dps proteins are instead classified as mini-ferritins and are widely distributed in the bacterial kingdom. They are smaller molecules made only of 12 subunits with a lower iron storage capacity [50,51] (Figure 1).

A common feature of all ferritins is that each subunit consists of a characteristic four-helical bundle (helices A-D), plus a fifth short helix E (not present in Dps proteins) pointing inside the protein cavity. Ferritin nanocages are able to self-assemble in a tetraeicosamer protein shell, and the dimers are the first intermediate in this pathway [50]. In the quaternary structure, channels are formed where subunits meet, located at the six four-fold and eight three-fold axes. Such pores connect the inner cavity to the outside and allow for the entry and the exit of iron ions and other small molecules [52].

Mammalian ferritins are typically made of two types of highly conserved subunits: the heavy chain H (21 kDa) and light one L (19 kDa). The H-chain subunit is able to oxidize Fe(II) to Fe(III), while the L one lacks catalytic activity but holds a microenvironment that facilitates iron nucleation and mineralization [53,54]. The gene for the H subunit is located on chromosome 11, whereas the gene encoding the L subunit is located on chromosome 19. Unlike the L gene, the H one undergoes transcriptional regulation dependent on the cell differentiation stage, changes in cell proliferation status, oncogenes, cytokines, and heme. The H:L ratio depends on tissue type and developmental stage. Acidic isoforms contain a greater number of H chains and are characteristically found in cardiac tissue. Basic isoforms are found in the liver, spleen, and plasma.

Ferritins are normally intracellular proteins. They localize mainly in the cytosol, and their physiological function is to store the iron in the form of nontoxic highly insoluble Fe(OH)_3_(H_2_O)_3_. Nevertheless, ferritins can also be detected in human serum [55] and internalized by cells through the transferrin receptor TfR1 (CD71) that recognizes the H subunit [56]. This applies also to mixed H/L-chain ferritin, as CD71 is able to recognize even a single subunit of the H-chain over 24. CD71 is an integral membrane homodimeric glycoprotein that mediates the uptake of transferrin-iron complexes. It binds to diferric transferrin at the cell surface and is internalized by chlatrin-dependent endocytosis. Diferric iron is subsequently released from transferrin due to the acidic endosomal pH. The transferrin receptor is mainly expressed on hepatocytes, myocytes, basal keratinocytes, endocrine pancreas, spermatocytes, and the erythroid precursor [57] and is abnormally expressed in many types of cancer. Given the high iron requirement of tumor cells, TfR1 can affect proliferation, migration, and invasion rates, as well as apoptosis and metastasis [58]. TfR1-mediated internalization makes ferritin an ideal candidate to target TfR1-overexpressing cancer cells [59].

Given the nanocage architecture of H ferritin that allows for easy encapsulation of a variety of drugs, it is not surprising that a lot of attention has been paid to the use of ferritins for a broad range of applications that span from imaging and diagnostics to chemotherapeutic delivery. For example, ferritin-based nanocages have been loaded with anthracyclines like doxorubicin, one of the most used drugs in cancer treatment, which stops cell proliferation by blocking isomerase 2 [60,61,62]. These studies showed that ferritin nanocarriers possess high loading efficiency and controlled drug release properties. In addition, ferritin nanocarriers possess excellent biodegradability, do not activate inflammatory or immunological responses, are able to passively penetrate the tumor tissues via Enhanced Permeability and Retention (EPR) effects, and exhibit blood-brain barrier (BBB)-traversing ability. Similar results were obtained by loading ferritin with other molecules with anticancer activity. Cisplatin, for instance, can be encapsulated within the central cavity of ferritin for targeted cancer treatments. Pt-based compounds are widely used in clinics for the treatment of several types of cancer, but they show significant side effects. Delivery systems like ferritin improve their overall biocompatibility, half-time life in the bloodstream, and target selectivity [63,64].

Mammalian Fts have also been used to encapsulate photosensitizers [65] to be used for the photodynamic treatment of cancer. The encapsulation allows to overcome targeting issues, providing an enhanced safety profile. Given the versatility of ferritins, it is not surprising that their applications are not limited to cancer treatment. In fact, they have also been used for diagnostics by encapsulating molecules or metals suitable for optical imaging [66], for catalysis [67], and even to trap entire proteins [68].

The encapsulation of the cargoes within the Ft cage is mainly carried out by the pH jump method: ferritin tetraeicosamer undergoes disassembly at extreme pH values (pH = 2.5 or 13.0), and then the protein is reassembled in the presence of the selected drug, restoring neutral pH. The main problem of this protocol is that extreme pH values can damage both the protein cage and the cargo, significantly reducing the efficiency of the encapsulation process. This process, in fact, is not fully reversible, leading to the formation of amorphous aggregates. The main drawback of the pH jump method is that only 50–60% of disassociated protein molecules could reassemble into a properly folded protein nanocage [69].

Alternative approaches, which involve the use of denaturing agents such as 8 M urea, do not substantially improve the incorporation efficiency, even leading to an excessive waste of the drug [64,70]. More recently, a new technique based on high hydrostatic pressure (HHP) has been developed to reversibly change protein conformation [71]. According to this method, ferritin solutions are pressurized at 500 MPa for 16 h in the presence of doxorubicin, thus allowing a good rate of encapsulation. None of the methods described above are suitable for incorporating therapeutic proteins inside the ferritin cage. Like ferritin, therapeutic proteins are also susceptible to loss of structure and function at extreme pH or in the presence of denaturing agents. Even high pressures do not guarantee the formation of sufficiently wide openings to allow the passage of large molecules as peptides and therapeutic proteins. Given these difficulties, researchers’ attentions have shifted to the development of alternative ferritins that could be able to associate/dissociate under physiological conditions.

Considering that noncovalent interactions are one of the main factors in stabilizing quaternary structures of proteins, a first approach was to identify, by mutagenesis, the key amino acid residues present at the subunit interfaces involved in ferritin self-assembly and stability [72,73]. Although some “switching residues” of oligomerization have been identified, no human ferritin able to associate and dissociate under physiological conditions has been developed so far.

Chen H. et al. [74] showed that the cleavage of the last 23 amino acids at the carboxyl terminal of human H ferritin alters the interfaces at the quaternary symmetry axes of native protein, allowing it to dissociate at milder pH values (pH 4). This mutant certainly allows to extend the choice of cargos but would not improve the recovery of properly assembled ferritin.

In this scenario, the ferritin from Archaeoglobus fulgidus, a strictly anaerobic, hyperthermophilic, marine archaeon, appears to be particularly promising, having an association equilibrium mediated by the saline concentration of the medium.

## 3. Archaeoglobus Fulgidus Ferritin: An Ideal Candidate for Protein Encapsulation

**Figure 2:**
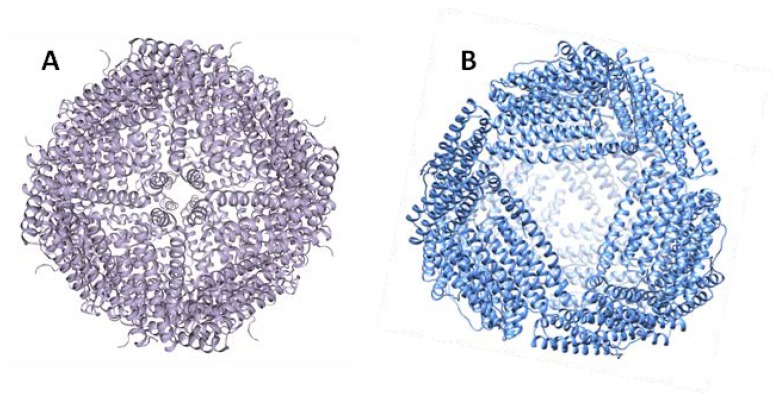
Tridimensional representation of (**A**) human H ferritin and (**B**) *Archaeoglobus fulgidus* ferritin. Despite the high degree of similarity between the secondary and tertiary structures, *A. fulgidus* ferritin (AfFt) and human ferritin differ in their quaternary structures. Human ferritin displays a tightly closed spherical shell with octahedral (4-3-2) symmetry; AfFt shows a roughly spherical shell with tetrahedral (2-3) symmetry containing four large pores.

Archaeal ferritin nanocages are taking center stage as privileged scaffolds for biotechnological applications due to their thermostability and exceptionally high yields when recombinantly expressed in *Escherichia coli*. Among them, *A. fulgidus* ferritin (AfFt) is gaining attention for its uncommon structure and association/dissociation properties that, at physiological pH, are only regulated by varying the ionic strength [75]. From a structural point of view, AfFt presents a tetrahedral assembly, which is unique in structural biology (Figure 2).

Unlike all the other known tetraeicosameric ferritins that assemble with octahedral (4-3-2) symmetry, the AfFt assembles in a roughly spherical shell with tetrahedral (2-3) symmetry. This unique quaternary structure is characterized by the presence on the protein surface of four large triangular pores (~45 Ả diameter) that can be considered as gates for the movement of ions and larger molecules in and outside the internal cavity.

**Figure 3:**
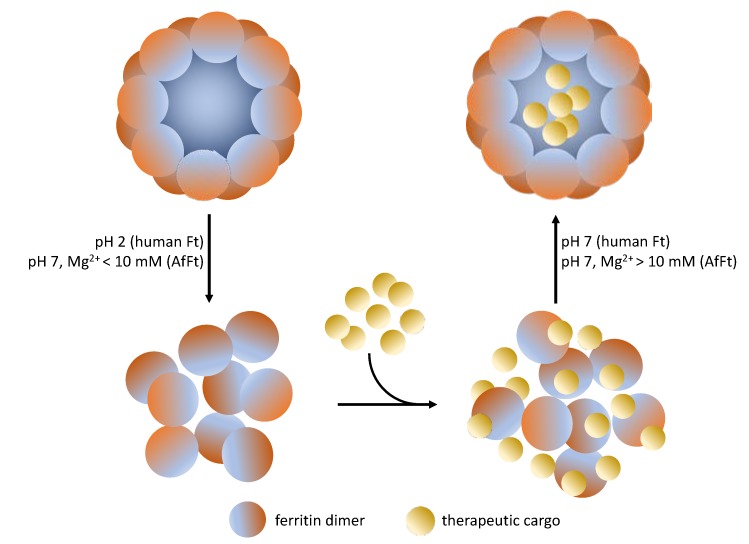
Encapsulation strategies of therapeutic cargoes in ferritin molecules. Human ferritin can be typically disassembled at very acidic pH values (pH 2) and reassembled in the presence of the given cargo, restoring neutral pH. Conversely, *A. fulgidus* ferritin is disassembled/reassembled at neural pH, just varying the concentration of divalent cations (i.e., MgCl_2_).

The main structural difference between the tetrahedral and octahedral symmetries relies on how the hexamers join to form the tetraeicosamer. In fact, in canonical ferritins such as human H ferritin, the hexamers are joined by four-fold symmetry elements. Conversely, AfFt hexameric assembly shows a two-fold symmetry. The type of final symmetry (four-fold or two-fold) is determined by specific amino acid residues at the hexamer–hexamer interface. Indeed, two specific residues (Lys-150 and Arg-151) are critical for the stabilization of the AfFt structure, as their replacement with alanine causes a switch from tetrahedral to closed octahedral symmetry [76]. Furthermore, AfFt assembly is strongly influenced by hydrophobic interactions, as observed in many other hyperthermophilic proteins [77]. For this reason, it is not surprising that the association/dissociation equilibrium is influenced by ionic strength. AfFt is associated in the form of a 24-subunit structure at high salt concentrations (Figure 3).

In fact, AfFt is completely associated with NaCl concentrations ≥500 mM. It has been observed that divalent cations such as Mg^2+^ mediate the same process at lower concentrations. The protein is completely closed when, for example, a concentration of MgCl_2_ greater than 10 mM is used. On the other hand, when low salt concentrations (NaCl < 100 mM or MgCl_2_ < 10 mM) are used, ferritin is present in the solution mainly as a dimer. The use of low concentrations of divalent cations to finetune the opening and closing of the protein cage has made it possible to exploit AfFt as a nanocarrier in physiological conditions. In addition, the electrostatic features of its inner surface make AfFt particularly suitable for the delivery of positively charged guests.

One of the first biotechnological applications of AfFt was its use for the synthesis of monodisperse gold nanoparticles. One of the greatest challenges in the synthesis of gold nanoparticles is to reduce the degree of polydispersity in order to get patterned structures with useful physical properties [78]. In this regard, the use of AfFt is particularly useful as it enables stable but also reversible interactions with gold nanoparticles. Pulsipher K.W. et al. [78] used this ferritin cage as a template for inorganic nanoparticle synthesis in more environmentally friendly conditions. Ferritin was able to incorporate preformed gold particles of 6 nm in diameter, restricting their growth to 8 nm. In addition, the presence of the wide openings on the protein surface provides a way to control Au nanoparticle chemistry in the solution, preventing their aggregation and bulk precipitation.

**Figure 4:**
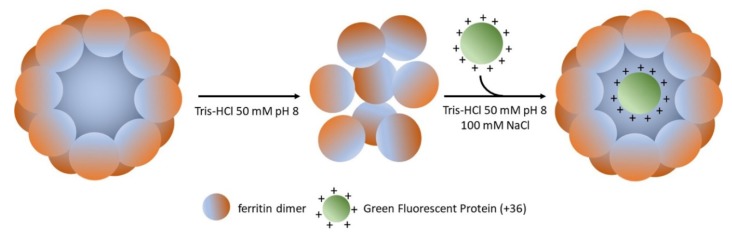
Encapsulation of supercharged (+36) green fluorescent protein (GFP) in *A. fulgidus* ferritin. Ferritin dissociates at low ionic strength, then supercharged GFP is spontaneously encapsulated in the protein cage (up to five molecules for tetraeicosamer), mediating its closure. 100 mM NaCl is required to avoid complexes’ precipitation.

Recently, AfFt was successfully loaded with enzymes inside the protein shell. Tetter and coworkers [79], as a proof of principle, were able to encapsulate up to five molecules of a supercharged (+36 positive charges) green fluorescent protein within the lumen cavity of AfFt. One of the most interesting aspects of this work is that green fluorescent protein (GFP (+36)) is itself capable of mediating the closure of the protein cage even at low ionic strength (Figure 4).

To broaden the cargo scope, the authors developed a strategy for incorporating enzymes into AfFt. Three different enzymes (the human carbonic anhydrase II36, the artificial (retro-)aldolase RA95.5-8F37, and Kemp eliminase HG3.17,38 evolved in their laboratory) were genetically fused to GFP(+36) and incorporated into the ferritin cage, just exploiting the ionic strength of the medium. These enzymes not only maintained their full catalytic activity but were also more resistant to proteolytic treatments. This work represents an interesting proof of concept, although it has some limitations. The enzyme-loaded nanoparticles developed in this paper have not been tested on cells to assess their uptake, probably because AfFt is a bacterial protein which cannot be selectively internalized by mammalian cells and could be immunogenic in vivo. Shuvaev et al. [80] were able instead to develop a superoxide dismutase (SOD)-loaded AfFt targeted to endosomes in endothelial cells. This work is particularly interesting because the authors show that it is possible to encapsulate moderately acidic proteins inside ferritin. SOD is covalently linked to a ferritin subunit, and the assembly of the nanoparticle is achieved by controlling the ionic strength of the medium rather than exploiting the electrostatic charge of the cargo itself. The targeting issue was faced developing an Ab/(Ft/SOD) nanocage. After encapsulating SOD inside the ferritin, the complex was crosslinked with PEGylated bis(sulfosuccinimidyl)suberate and subsequently conjugated with anti-caveolar plasmalemmal vesicle-associated protein (PLVAP) antibodies. In vitro and in vivo data showed that this tri-molecular complex was selectively delivered to caveolar endosomes blocking the proinflammatory effects of lipopolysaccharides in animals. However, given the complexity of the preparation of this trimolecular complex, it is difficult to imagine its use on a large scale.

Thus, although AfFt represents a very versatile tool for the encapsulation of therapeutic agents, what limits its use is the lack of selectivity towards animal cells.

## 4. *Archaeoglobus fulgidus*-Human Chimeric Ferritin for Targeted Protein Delivery

One of the main drawbacks of nanoparticle-mediated protein delivery is the selective targeting [81]. Although new proteins and peptides are constantly identified as potential drugs, the development of delivery systems continues to be a challenge mainly due to the difficulty to get a site-specific pharmacological action. Proteinaceous anticancer drugs are typically delivered through an EPR-mediated passive accumulation, which, however, reduces its therapeutic potential. A promising alternative is to associate the therapeutic protein with a protein ligand (e.g., an antibody) capable of recognizing specific antigens/receptors on the surface of tumor cells. Similarly, it is possible to engineer the protein nanoparticles to confer them the desired selectivity, as indeed it has been done in the case of AfFt. As previously discussed, this ferritin has the great advantage of opening and closing in physiological conditions, but unlike human H ferritin, it does not have intrinsic tumor-targeting properties.

**Figure 5:**
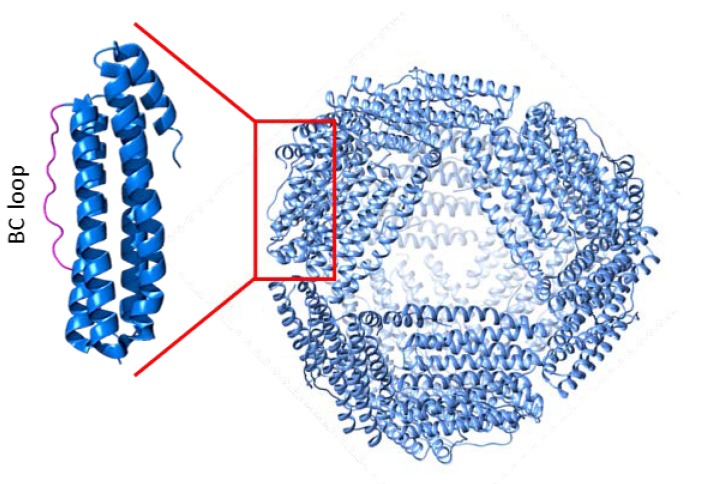
Tridimensional structure of *A. fulgidus*-human chimeric ferritin. Each *A. fulgidus* ferritin monomer was mutated by replacing the amino acid residues of the BC loop with the corresponding amino acids of human H ferritin. This modification allows the chimeric ferritin to be recognized by the TfR1 receptor.

One of the ways to make AfFt selective in targeting human cells is engineering its protein surface. The most logical change to carry out would be the one favoring its binding to the human ferritin H receptor. Recently, a chimeric A. fulgidus-human ferritin with an acquired selectivity towards the human TfR1 receptor has been developed [82]. TfR1 is human transferrin receptor 1 (also known as CD71) involved in iron supply by endocytosis upon binding of iron-loaded transferrin and ferritin. The study of this interaction is of paramount importance, not only from a physiological point of view, but also in view of the potential nanobiotechnological applications that are being developed for ferritin. In 2019, the Cryo-EM structure of the human ferritin–transferrin receptor 1 complex has been reported for the first time [83]. H-Ft binds the apical domain of the CD71 receptor through four specific contact regions in the ferritin BC loop. It can therefore be assumed that the presence of this specific recognition sequence could give AfFt the ability to bind TfR1. Additionally, de Turris et al. [83] showed that, by replacing 12 amino acids of the AfFt BC loop with the corresponding sequence of human H ferritin, a chimeric protein is obtained that holds the typical association/dissociation properties of AfFt while keeping the ability to selectively bind to the TfR1 receptor (Figure 5).

This protein, just like transferrin, is efficiently uptaken by HeLa cells and can be detected in the cytoplasm and in the perinuclear space, suggesting a typical clathrin-coated endocytosis pathway mediated by TfR1.

This chimeric ferritin represents a unique scaffold for incorporating therapeutic cargoes, including bioactive proteins, inside its cavity. This can be easily achieved either by assembly/disassembly process at neutral pH or by diffusion through the large triangular pores on the surface. Another advantage of this reversible assembly/disassembly dynamic is the ability to release the payload within the cytosol once ferritin is uptaken by the cell.

The first application of this chimeric ferritin was the delivery of cyt C (cytochrome C) to acute lymphoid leukemia cells [41]. Cyt C is a small basic protein capable of inducing apoptosis when released from the mitochondria into the cytosol, where it interacts with Apaf-1, thus activating the caspase cascade. Considering the dimensions and the net positive charge of cyt C, it is particularly suitable for being encapsulated in the chimeric ferritin. To date, different approaches have been attempted to selectively deliver this therapeutic protein to cancer cells. Cyt C, for example, was immobilized on mesoporous silica nanoparticles via intermediate linkers of disulfide bonds for redox-responsive intracellular drug delivery. A tumor-targeting tissue was faced linking to cyt C AS1411 aptamer, which targets and binds to nucleolin, a nucleolar phosphoprotein which is overexpressed on the surface of HepG2 cells [84]. In another study, cyt C was specifically targeted to glioma cells, exploiting the proton-coupled folate transporter, which is overexpressed in these cells [85]. A folate-receptor targeting amphiphilic copolymer (folic acid-PEG-poly(lactic-co-glycolic acid)) was attached to cyt C through a redox-sensitive bond. Kim S.K. et al. [86] instead developed a liposome-apolipoprotein A-I nanoparticle to deliver cyt C to non-small cell lung tumors. In this case cyt C was previously conjugated with a membrane permeable sequence peptide. The cyt C-loaded nanoparticle was further tailored with PEG–distearoylphosphatidylethanolamine-anisamide to enable specific targeting to the tumor site.

**Figure 6:**
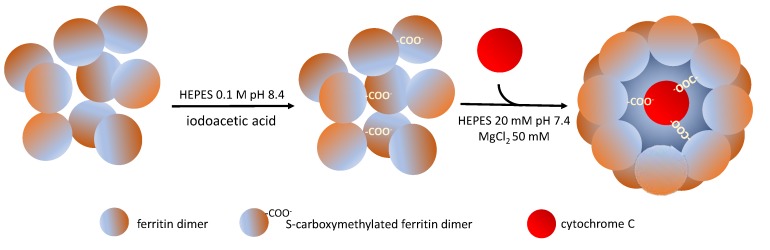
Encapsulation of cytochrome C in *A. fulgidus*-human chimeric ferritin. Ferritin dimers were previously S-carboxymethylated with iodoacetic acid to enhance the net negative charge of the inner cavity. Reassembly of the protein cage was then achieved at neutral pH in the presence of cytochrome C and divalent cations.

All the cyt C delivery systems described above made use of complex nanoparticles. An alternative and certainly simpler approach to selectively deliver cyt C to target cells is the use of chimeric *A. fulgidus*-human ferritin. The inner cavity of this protein was designed by inserting a cysteine residue for each subunit that makes possible to bind, even covalently, cargoes such as fluorophores, nucleic acids, proteins, or therapeutic peptides. In addition, the same site allows the cavity to be functionalized, finetuning the internal charge of the cage according to the chemical-physical properties of the substance to be encapsulated. Macone et al. [41] showed that this chimeric ferritin is indeed able to encapsulate cyt C and to deliver it to acute lymphoid leukemia cells expressing high levels of TfR1. This is noteworthy as these cells are practically resistant to any type of known transfection. To improve the encapsulation efficiency of cyt C within the protein shell, the cysteine residues were carboxymethylated to increase the net negative charge of the internal cavity (Figure 6). The addition of negative charges, however, could affect the association equilibrium of the chimeric ferritin. The authors have established that, in order to have a correct assembly of the protein, it is necessary to derivatize no more than 50% of the available cysteines. The ferritin-cyt C complex was efficiently internalized by tumor cells and, once released, cyt C was able to induce apoptosis.

## 5. What Is the Next Step?

The work on chimeric ferritins is in its infancy. In order to extend studies on in vivo systems, it is necessary to address the possible immunogenicity of the chimeric construct. Should it turn out to be immunogenic, this ferritin will have to be modified on the surface to allow its use on humans. Nevertheless, there is a wide range of therapeutic proteins and peptides that can be encapsulated to assess their cellular effect. By genetic engineering, it is also possible to insert recognition sequences for surface receptors other than TfR1, thus targeting different tissues or cellular types.
